# Diffuse Alveolar Hemorrhage Associated With Anti-PL-7 Antisynthetase Syndrome: A Case Report

**DOI:** 10.1155/crpu/3715449

**Published:** 2025-01-17

**Authors:** Paul Shiu, Shannon Iriza, Steven Templeton

**Affiliations:** Prisma Health, University of South Carolina–School of Medicine, Columbia, South Carolina, USA

## Abstract

**Background:** Diffuse alveolar hemorrhage (DAH) is a potentially life-threatening condition which can present with hemoptysis, diffuse alveolar infiltrates, anemia, and hypoxic respiratory failure. Antisynthetase syndrome (AS) is a rare autoimmune disorder most often characterized by nonerosive arthritis, proximal muscle weakness with elevated muscle enzymes, Raynaud's phenomenon, hyperkeratosis of the digits (mechanic's hands), and interstitial lung disease. According to large population studies, AS has an annual incidence of 0.56 per 100,000 persons and prevalence of 9 per 100,000. The most common autoantibody is anti-aminoacyl-transfer RNA synthetase for histidine (anti-Jo-1) with a reported prevalence of 20%–30%, whereas anti-Pl-7 (for threonine) accounts for less than 5% of all autoimmune myositis. Specific myositis autoantibodies determine clinical phenotype. PL-7 is characterized by interstitial lung disease, myositis, and arthritis. Autoimmune myositis, specifically AS, is a rare cause of DAH. Herein, we describe the first reported case of PL-7-associated AS with DAH.

**Case Presentation:** A 41-year-old female presented with worsening shortness of breath and hemoptysis. Laboratory studies included a hemoglobin of 10.5 g/dL, mildly elevated liver enzymes, and a creatine phosphokinase (CPK) of nearly 4000 U/L. CT of the chest showed diffuse ground glass opacities bilaterally. Serial aliquots of the bronchoalveolar lavage (BAL) fluid revealed progressively hemorrhagic return and histopathologic analysis consistent with DAH. Other concurrent causes of DAH were ruled out.

**Conclusion:** Although rare, AS should be considered a cause of DAH, particularly in patients presenting with symptoms of muscle weakness and arthritis or with evidence of mechanic's hands.

## 1. Background

Diffuse alveolar hemorrhage (DAH), caused by a disruption to the alveolar–capillary basement membrane, is a potential life-threatening condition which can present with hemoptysis, diffuse alveolar infiltrates, anemia, and hypoxic respiratory failure [1]. There are many causes of DAH, with autoimmune disease being one of them. The most common autoimmune causes of DAH are from systemic vasculitides including antineutrophil cytoplasmic antibody (ANCA)–associated vasculitides (AAV) at 25% [2], antiglomerular basement membrane (GBM) disease at 5%–10% [3], and IgA vasculitis at 0.8%–5% [4]. Contrary to popular belief, it is significantly less common for lupus at < 2% [5], antiphospholipid syndrome at 2% [6], and other autoimmune conditions to be associated with DAH. Infections, such as bacterial or viral pneumonia; medications, most commonly anticoagulants or antiplatelet medications; pulmonary edema; acute respiratory distress syndrome; and lung injuries attributed to trauma, surgery, or radiation therapy are also possible causes. Radiographically, DAH typically shows bilateral opacities, which are nonspecific [1].

Antisynthetase syndrome (AS) is a rare autoimmune disorder, with an annual incidence of 0.56 per 100,000 persons and prevalence of 9 per 100,000, most often characterized by nonerosive arthritis, proximal muscle weakness with elevated muscle enzymes, Raynaud's phenomenon, hyperkeratosis of the digits (mechanic's hands), and interstitial lung disease (ILD) [7, 8]. The pathophysiology of AS is thought to be caused by a combination of genetic factors of which HLA-DRB1 is thought to be the strongest genetic risk factor, environmental triggers such as UV light exposure and vitamin D levels, and infections triggering autoimmunity through epitope spreading and molecular mimicry [9]. This autoimmune–inflammatory milieu may then lead to the development of autoantibodies against one of the aminoacyl-transfer RNA (tRNA) synthetases, listed in order of frequency, such as anti-aminoacyl-transfer RNA synthetase for histidine (anti-Jo-1) (histidine), anti-PL-12 (alanine), anti-PL-7 (threonine), anti-OJ (isoleucine), anti-KS (asparagine), and anti-EJ (glycine) [8]. The most common autoantibody is anti-Jo-1 with a reported prevalence of 20%–30%, whereas anti-Pl-7 (for threonine) accounts for less than 5% of all autoimmune myositis [8]. Specific autoantibodies determine clinical phenotype. Anti-PL-7 is characterized by ILD, myositis, and arthritis [8]. Compared to anti-Jo-1, anti-PL-7 has a higher incidence of ILD and lower incidence of myositis. Nonerosive arthritis and mechanic's hands are seen more often with anti-Jo-1 whereas heliotrope rash has been described more in anti-PL-7 [10]. Up to 70% of patients with AS develop ILD [7]. The time course of the development of ILD is variable and may precede myositis, present simultaneously, or develop later in the disease course. The ILD associated with AS is most commonly nonspecific interstitial pneumonia and/or organizing pneumonia; however, usual interstitial pneumonia has also been seen [8]. In the literature, the coexistence of anti-Ro52 (SSA) antibodies in patients with AS has been associated with worse pulmonary outcomes. More medications were needed to control the disease process, and remission was less common in these patients [11]. Autoimmune myositis, specifically AS, is a rare cause of DAH; however, it has been described in the literature. Herein, we describe the first reported case of PL-7 associated with DAH.

## 2. Case Presentation

This is a 41-year-old female with recently diagnosed anti-PL-7 AS who also tested positive for anti-Ro52 (SSA). At the time of her original diagnosis, she had Raynaud's phenomenon, arthritis, myositis, and ILD. At presentation in follow-up, she was afebrile, normotensive, tachycardic with a heart rate of 124 beats per minute, tachypneic with a respiratory rate of 32 breaths per minute, and saturating 88% on 2-L nasal cannula. She was in moderate respiratory distress with dyspnea on exertion. She had crackles bilaterally on auscultation. Cutaneous manifestations included a violaceous erythematous eruption of the upper eyelids, known as heliotrope rash, as well as along the upper back and anterior chest, known as shawl sign and v-sign, respectively. She did not have lower extremity edema, mechanic's hands, petechiae, purpura, or synovitis.

**Figure 1:**
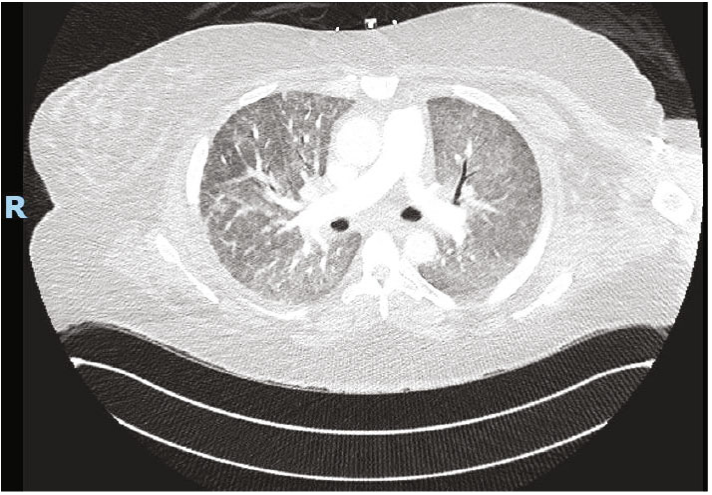
Computed tomography of the chest with angiography. Diffuse ground glass and reticular opacities are present bilaterally with areas of subpleural sparing. There is also mild bronchiectasis. There is no air trapping, honeycombing, or head cheese sign.

**Figure 2:**
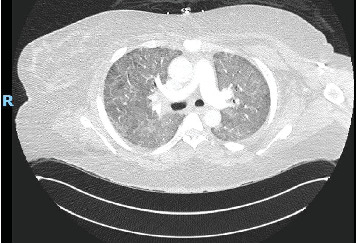
Computed tomography of the chest with angiography. Worsening diffuse ground glass opacities are present concerning for edema, inflammation, or hemorrhage. There is a similar degree of bronchiectasis compared to prior.

**Figure 3:**
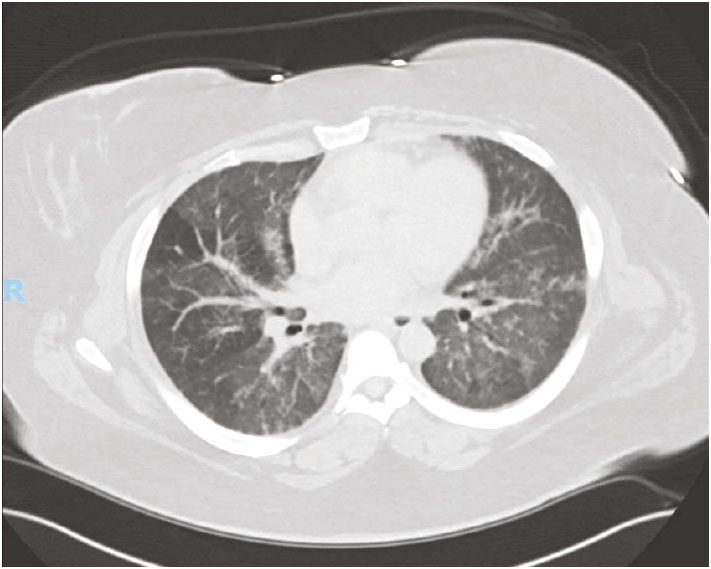
Computed tomography of the chest without contrast. Decreased ground glass opacities bilaterally, which is consistent with resolving pulmonary hemorrhage.

She was being treated with 80 mg of prednisone and Imuran 50 mg daily when she presented to the pulmonary office with worsening cough, dyspnea on exertion, and hemoptysis. She was subsequently admitted to the hospital for further evaluation and treatment. Computed tomography (CT) angiography of the chest was negative for pulmonary embolism but did reveal diffuse bibasilar predominant ground glass opacities, reticulation, and mild bronchiectasis without honeycombing (Figure 1). This represented an interval worsening compared to prior imaging (Figure 2). Complete blood cell count and comprehensive metabolic panel showed a white count of 21.0 × 10E3/*μ*L, hemoglobin of 10.5 g/dL, and mildly elevated transaminases (AST 55 IU/L and ALT 70 IU/L). Her CPK (3980 U/L) and aldolase (55.2 U/L) were both significantly elevated. Urinalysis was unremarkable with bland sedimentation. SARS-CoV2 PCR testing was also negative. ANCA and ANA via IFA were negative. She subsequently underwent bronchoscopy for airway inspection and bronchoalveolar lavage. The initial BAL fluid was bloody. Serial aliquots became progressively bloodier consistent with DAH. Histopathologic analysis revealed hemosiderin-laden macrophages. A pneumonia pathogen PCR panel, gram stain and culture, AFB culture, fungal culture, and *Pneumocystis jirovecii* direct fluorescence antibody staining on the BAL fluid all returned negative. Once the diagnosis of DAH was confirmed and infection was excluded, in the setting of escalating oxygen requirements with noninvasive positive pressure ventilation, the patient received pulse-dose steroids with 1 g of methylprednisolone for 3 days. She was then converted to 1 mg/kg PO prednisone. In addition, she received 2 g/kg of IVIG and induction therapy with rituximab of 1 g for two doses given 14 days apart. On this regimen, the patient's CPK improved to 617 and she was able to return to her baseline 2-L nasal cannula after several days. Around the time of hospital admission, her DLCO was 35%. She was followed on an outpatient basis and her azathioprine was uptitrated; however, this was subsequently changed to mycophenolate mofetil due to gastrointestinal intolerance. She has been maintained on mycophenolate mofetil 2 g per day, Plaquenil 400 mg daily, and monthly IVIG as well as rituximab 1 g two doses every 6 months. She has been able to taper her prednisone 120 mg down to 10 mg per day without a disease flare. Follow-up CPK and aldolase levels have normalized. CT of the chest demonstrated decreased diffuse ground-glass opacities particularly at the lung bases (Figure 3) compared to prior imaging. Pulmonary function tests (PFTs) prior to admission revealed an FVC of 1.85 L (57% of predicted), an FEV1 of 1.61 L (67%), and diffusion capacity of 12.38 mL/min/mmHg (56%). PFTs a few months after hospital discharge and 6 months after her previous testing revealed an FVC of 1.75 L (53%), an FEV1 of 1.63 L (61%), and diffusion capacity of 9.44 mL/min/mmHg (43%). The decrease in FEV1 and FVC may be attributed to her 25-kg weight gain which is likely from steroid administration and her sedentary state. Stability and clinical improvement rather than continued decline in her respiratory status, as can be seen in those with the PL-7 antibody, can be attributed to appropriate medical therapy.

## 3. Discussion and Conclusions

Autoimmune myositis, specifically AS, is a rare cause of DAH. While the causative mechanism remains to be elucidated, though thought to be a consequence of alveolar-basement membrane related damage due to inflammatory changes at the interface, there have been a few cases of concurrent DAH with AS steadily reported suggesting an association of clinical consequence [12–15]. The earliest reported case was by Schwarz and colleagues who described two cases of pulmonary capillaritis associated with inflammatory myositis—one case had anti-Jo-1 antibody. Both cases were described to have severe proximal myopathy, elevated serum creatine phosphokinase levels, and inflammatory muscle biopsies. Nearly a decade later, Kummerfeldt et al. discussed a case of a previously healthy 32-year-old woman with anti-PL-1- associated AS who presented with progressively worsening shortness of breath in the setting of DAH. Muscle enzymes and strength testing were normal. The next subsequent case reports would follow 8 and 9 years later. Vargas-Gutierrez, Solis-Jimenez, and Callejas described another case of DAH in the setting of anti-PL-12 and Ro52 antibodies, a 40-year-old male who initially presented with nonspecific dry cough and moderate dyspnea, mechanic's hands, and subclinical myositis on electromyography. The other study by Samuel et al. reported a 44-year-old male with anti-Jo-1 antibody demonstrating the triad of dyspnea, hemoptysis, and diffuse patchy opacities on radiographs along with proximal muscle weakness. Taken together, this case highlights the importance of having a high clinical suspicion of AS when considering a differential for DAH, particularly in patients presenting with compatible clinical features such as muscle weakness, arthritis, characteristic skin rashes, and Raynaud's phenomenon. Of note, transaminitis can occur in muscle breakdown and could be a clue to an underlying myopathy. As previously described, different myositis-specific antibodies have prognostic implications for the type of organ involvement and the clinical course. In addition, coexistent Ro-52 positivity in antisynthetase patients has suggested development of more aggressive lung disease which helps to guide physicians as to the recommended intervals for monitoring for progression of lung disease as well as treatment choices [11]. Rituximab and cyclophosphamide are both acceptable induction agents. As demonstrated in the RECITAL trial, patients with connective tissue disease ILD treated with rituximab compared to cyclophosphamide also demonstrated an increase in FVC however with less adverse side effects [16]. Cyclophosphamide was not favored due to its risk of adverse events such as infection, malignancy, infertility, hemorrhagic cystitis, and cytopenias [17]. IVIG is an immunomodulatory rather than an immunosuppressive medication and can therefore be safely combined with other induction agents and has a rapid onset of action which makes it an ideal agent for adjuvant treatment in patients with impending respiratory failure.

## Data Availability

The data that support the findings of this study are available on request from the corresponding author. The data are not publicly available due to privacy or ethical restrictions.

## Nomenclature

ANA: antinuclear antigen
ANCA: antineutrophil cytoplasmic antibody–associated vasculitides
BAL: bronchoalveolar lavage
CPK: creatine phosphokinase
DAH: diffuse alveolar hemorrhage
GBM: antiglomerular basement membrane disease
IFA: immunofluorescence assay
ILD: interstitial lung disease
